# Core regulatory components of the PHO pathway are conserved in the methylotrophic yeast *Hansenula polymorpha*

**DOI:** 10.1007/s00294-016-0565-7

**Published:** 2016-01-21

**Authors:** Ying Zhou, Naoya Yuikawa, Hiroki Nakatsuka, Hiromi Maekawa, Satoshi Harashima, Yoichi Nakanishi, Yoshinobu Kaneko

**Affiliations:** Yeast Genetic Resources Laboratory, Graduate School of Engineering, Osaka University, Osaka, 565-0871 Japan; Department of Biotechnology, Graduate School of Engineering, Osaka University, Osaka, 565-0871 Japan; Department of Biological Mechanisms and Functions, Graduate School of Bioagricultural Sciences, Nagoya University, Nagoya, 464-8601 Japan; Division of Applied Microbial Technology, Graduate School of Engineering, Sojo University, Kumamoto, 860-0082 Japan

**Keywords:** PHO pathway, *H.* *polymorpha*, Regulatory gene, Phosphate homeostasis, Diversity

## Abstract

To gain better understanding of the diversity and evolution of the gene regulation system in eukaryotes, the phosphate signal transduction (PHO) pathway in non-conventional yeasts has been studied in recent years. Here we characterized the PHO pathway of *Hansenula polymorpha*, which is genetically tractable and distantly related to *Saccharomyces cerevisiae* and *Schizosaccharomyces pombe*, in order to get more information for the diversity and evolution of the PHO pathway in yeasts. We generated several *pho* gene-deficient mutants based on the annotated draft genome of *H.* *polymorpha* BY4329. Except for the *Hppho2*-deficient mutant, these mutants exhibited the same phenotype of repressible acid phosphatase (APase) production as their *S.* *cerevisiae* counterparts. Subsequently, *Hppho80* and *Hppho85* mutants were isolated as suppressors of the *Hppho81* mutation and *Hppho4* was isolated from *Hppho80* and *Hppho85* mutants as the sole suppressor of the *Hppho80* and *Hppho85* mutations. To gain more complete delineation of the PHO pathway in *H.* *polymorpha*, we screened for UV-irradiated mutants that expressed APase constitutively. As a result, three classes of recessive constitutive mutations and one dominant constitutive mutation were isolated. Genetic analysis showed that one group of recessive constitutive mutations was allelic to *HpPHO80* and that the dominant mutation occurred in the *HpPHO81* gene. Epistasis analysis between *Hppho81* and the other two classes of recessive constitutive mutations suggested that the corresponding new genes, named *PHO51* and *PHO53*, function upstream of *HpPHO81* in the PHO pathway. Taking these findings together, we conclude that the main components of the PHO pathway identified in *S.* *cerevisiae* are conserved in the methylotrophic yeast *H.* *polymorpha,* even though these organisms separated from each other before duplication of the whole genome. This finding is useful information for the study of evolution of the PHO regulatory system in yeasts.

**Electronic supplementary material:**

The online version of this article (doi:10.1007/s00294-016-0565-7) contains supplementary material, which is available to authorized users.

## Introduction

To adapt to environmental changes and survive in various conditions, microorganisms have evolved complex pathways of signal transduction. A phosphate-responsive signaling pathway, known as the PHO pathway, regulates the genes required to maintain proper phosphate homeostasis in the budding yeast *Saccharomyces cerevisiae* and is the best-studied example of phosphate homeostasis in eukaryotes (reviewed in Ljungdahl and Daignan-Fornier 2012; Oshima 1997; Yadav et al. 2015). Because inorganic phosphate (Pi) is an essential nutrient for all organisms, it is thought that the PHO pathway is likely to be conserved in other eukaryotes at least to some extent and to be a good model system for studying the diversity and evolution of the regulatory system in eukaryotes. In *S.* *cerevisiae*, under the condition of high extracellular phosphate, the transcriptional activator Pho4 is phosphorylated by the cyclin-dependent kinase–cyclin (CDK–cyclin) complex Pho85–Pho80, transported from the nucleus to the cytoplasm by Msn5, and prevented from interaction with the transcription factor Pho2. As a result, phosphate-repressed genes, such as *PHO5* and *PHO84*, are not expressed under this condition. When the extracellular phosphate concentration is sufficiently low, by contrast, the CDK inhibitor (CKI) Pho81, which is activated by inositol heptakisphosphate (IP_7_, synthesized by Vip1), inhibits the activity of the Pho85–Pho80 complex, enabling Pho4 to localize in the nucleus and induce the expression of phosphate-repressed genes in collaboration with Pho2 (Kaffman et al. 1994, 1998a, b; Lee et al. 2007; Ogawa et al. 1995; Schneider et al. 1994). Recently, Pho92 containing a YTH domain has been reported to regulate *PHO4* mRNA stability and to be involved in phosphate and glucose metabolisms (Kang et al. 2014). Although many components of the PHO pathway have been well characterized, the molecular mechanism underlying phosphate sensing remains unclear.

Recently, the PHO pathway has been studied in non-conventional yeasts in order to gain better understanding of the diversity and evolution of the gene regulation system in eukaryotes. For example, many regulatory components of the PHO pathway are conserved in *Candida glabrata*, although *CgPHO2* is not required for transcriptional induction of the phosphate starvation genes (Kerwin and Wykoff 2009). *Candida glabrata* has also lost the gene *PHO5*, which encodes a phosphate-repressible acid phosphatase (APase), and instead has evolved the gene *CgPMU2*, which encodes a broad-range phosphate starvation-regulated acid phosphatase that functionally replaces *PHO5* (Orkwis et al. 2010). On the other hand, the PHO pathway of the fission yeast *Schizosaccharomyces pombe* includes no genes orthologous to *ScPHO81*, *ScPHO2* and *ScPHO4*, and its orthologs of *ScPHO80* and *ScPHO85* are not involved in the PHO pathway. In addition, the SpPho7 transcriptional activator for the APase gene (*Sppho1*) is a zinc-finger-type activator that differs from the helix–loop–helix type of the ScPho4 activator (Henry et al. 2011). Moreover, this *S.* *pombe* PHO pathway is cross-regulated at the SpPho7 site by phosphate and adenine (Estill et al. 2015).

The PHO pathway has also been studied in the filamentous fungi *Neurospora crassa* and *Aspergillus nidulans* since the 1960s. The PHO pathway of *N.* *crassa* is known to comprise the genes *nuc*-*1*, *preg*, *pgov* and *nuc*-*2*, which correspond to *ScPHO4*, *ScPHO80*, *ScPHO85* and *ScPHO81*, respectively (Kang and Metzenberg 1990; Littlewood et al. 1975; Toh-e and Ishikawa 1971). In *A.* *nidulans*, by contrast, the genes that are homologous to *ScPHO80* and *ScPHO4* are involved in the PHO pathway, but those homologous to *ScPHO81* and *ScPHO85* are not (Wu et al. 2004). In addition, a recent study on the PHO system of *Cryptococcus neoformans* reported that *CnPHO4*, *CnPHO80*, *CnPHO85* and *CnPHO81* are PHO regulatory genes (Toh-e et al. 2015). Thus, the cyclin–CDK–CKI complex of core regulatory components in the PHO pathway is not always conserved, although the PHO pathway itself is conserved, even in fungi that are evolutionarily distantly related to *S.* *cerevisiae*.

The methylotrophic yeast *H.* *polymorpha* is genetically tractable and phylogenetically positioned between *S.* *cerevisiae* and the fission yeast *S.* *pombe* or filamentous fungi (Dujon 2010; Dujon et al. 2004). It diverged from a common ancestor with *S.* *cerevisiae* about a billion years ago, and did not undergo whole-genome duplication. To our knowledge, there have been no studies on its PHO pathway, although *HpPHO1* was identified to encode an APase more than 10 years ago (Phongdara et al. 1998). Here, we report characterization of the PHO pathway in *H.* *polymorpha.* Using a combination of comparative genomic analysis and classic genetics, we confirm that the PHO pathway consists of *HpPHO81*, *HpPHO80*, *HpPHO85* and *HpPHO4* genes. The genetic roles and positions of these genes in the PHO system are the same as the corresponding genes of *S.* *cerevisiae*. In addition, mutants exhibiting constitutive APase expression (termed the Pho^C^ phenotype) were isolated and classified into four groups comprising three groups of recessive mutations and one group of dominant mutations. One of the three recessive Pho^C^ mutations was found to be allelic to *Hppho80*, whereas the dominant mutation was allelic to *HpPHO81*. Epistasis analysis of the remaining two groups of recessive mutations with the *Hppho81* mutation indicated that the two corresponding genes both function upstream of *HpPHO81* in the PHO pathway. We conclude that the main components of the PHO pathway are conserved in *H.* *polymorpha,* although this yeast separated from *S.* *cerevisiae* before duplication of the whole genome.

## Materials and methods

### Strains, plasmids and media

The yeast strains and plasmids used in this study are listed in Table 1. Strain BY4329 was used as the wild-type strain for isolation of APase mutants. Strain NY-1 was used for isolation of suppressors of *Hppho81* mutation. *H.* *polymorpha* gene disruptants were constructed by using a zeocin- or hygromycin-resistance gene, or *HpURA3* cassette. Disruption of genes was confirmed by PCR. To construct plasmids YZ3 and YZ6, the *HpPHO81* ORF and *ScPHO81* ORF were first amplified by PCR and then inserted into plasmid BYP7151 digested by *Bam*HI and *Hin*dIII, respectively, via an In-Fusion Cloning kit (Takara Bio Inc., Japan). To construct plasmid YZ14, the *HpPHO4* ORF plus 500-bp upstream and 300-bp downstream sequences was amplified by PCR, and inserted into the *Sma*I restriction site of pFL26. The *HpPHO80*- and *HpPHO85*-expression plasmids (YZ77 and YZ78, respectively) were constructed in the same way. For sequence analysis of *HpPHO81*^*C*^ alleles, each *HpPHO81*^*C*^ allele was amplified by PCR and cloned into the *Bam*HI–*Hin*dIII gap of pUC19. The PCR primers used to generate strains and plasmids are shown in Table S1.

**Table 1:** Strains and plasmids used in this study

Strain or plasmid	Genotype or description	Source
*H.* *polymorpha*		
BY4329	*leu1*-*1*	NBRP^a^
BY4330	*ura3*-*1*	NBRP^a^
KYC638	*ura3*-*1 leu1*-*1*	Lab stock
KYC1389	*pho80*-*2 ade11*-*1*	This study
KYC1390	*pho85*-*8 ura3*-*1*	This study
KYC1404	*pho85*-*8 leu1*-*1*	This study
NY-1	*pho81*Δ*::URA3 ade11*-*1 ura3*-*1*	This study
H76-1B	*pho81*Δ*::URA3 leu1*-*1 ura3*-*1*	This study
HPH27-3	*pho80*Δ*::zeo ura3*-*1*	This study
YZS216	*pho85*Δ*::hphNT leu1*-*1 ade8*-*1*	This study
HPH7-26	*pho4*Δ*::zeo ura3*-*1*	This study
HPH26	*pho2*Δ*::zeo ura3*-*1*	This study
YZS28	*pho4::pREMI*-*Z leu1*-*1*	This study
YZS135	*pho4*-*1 ura3*-*1*	This study
*S.* *cerevisiae*		
BY22357	*MATα pho3 pho81*∆*::LEU2 leu2 ura3 trp1 his3*	NBRP^a^
Plasmid		
N3	*HpPHO81* in pUC19	This study
BYP5153	*ScPHO81* in YEp13	NBRP^a^
BYP7151	pBP-G2; *TEF1*-*PGK1* bidirectional promoter	Partow et al. (2010)
YZ3	*HpPHO81* ORF in BYP7151	This study
YZ6	*ScPHO81* ORF in BYP7151	This study
YZ14	*HpPHO4* in pFL26	This study
YZ77	*HpPHO80* in pFL26	This study
YZ78	*HpPHO85* in pFL26	This study
pAP4	*HpPHO81* ^*C*^ allele of AP4 in pUC19	This study
pAP5	*HpPHO81* ^*C*^ allele of AP5 in pUC19	This study
pC12	*HpPHO81* ^*C*^ allele of C12 in pUC19	This study
pC51	*HpPHO81* ^*C*^ allele of C51 in pUC19	This study

^a^National BioResource Project-Yeast, http://yeast.lab.nig.ac.jp/nig/index_en.html

Yeast strains were grown in nutrient medium containing 200 mg/l of adenine (YPAD), synthetic dextrose medium (SD), or minimal high-Pi and low-Pi media supplemented with appropriate amino acids when needed (Sherman 1991; Toh-e et al. 1973). Mating and sporulation of *H. polymorpha* were induced on 2.5 % maltose and 0.5 % malt extract medium (MAME) plates at 30 °C. For drug resistance selection, the plates were supplemented with hygromycin B (150 µg/ml, Wako Pure Chemical Industries Ltd., Japan) or Zeocin™ (100 µg/ml, Invitrogen Corp., USA). *E.* *coli* DH5α was cultured in LB broth (Miller 1972) and used for plasmid DNA preparation. For solid media, 2 % agar was added. The growth temperature for yeast and bacteria was 37 °C unless stated otherwise.

### Genetic analysis and transformation

Diploid cells were constructed by crossing strains that had auxotrophic mutations complementary to each other on MAME plates. After incubation at 30 °C for 1 day, the mating culture was transferred to an SD plate, and only diploid cells were selected as a prototrophic culture. For sporulation, the resulting diploid cells were incubated on a new MAME plate for 2 days at 30 °C. Tetrads were dissected by a SporePlay™ micromanipulator (Singer Instruments Co. Ltd., UK). Yeast transformation was performed with a Frozen-EZ Yeast Transformation II kit (Zymo Research Inc., USA). For *E.* *coli* transformation, *ECOS*™ Competent *E.* *coli* DH5α (Nippon Gene Co. Ltd., Japan) was used according to the manufacturer’s instructions.

### Genome sequencing and gene annotation

The draft genome sequence of BY4329 was determined and submitted to the DNA Data Bank of Japan (DDBJ), as described by Maekawa and Kaneko (2014). Its BioProject ID is PRJDB3035. Predicted CDS data of the draft genome sequence were obtained by using In Silico Molecular Cloning (IMC) version 5.1 (in silico Biology Inc., Japan) and query amino acid sequences from three yeast amino acid databases (*S.* *cerevisiae*, *Ogataea parapolymorpha* and *S.* *pombe*). Gene annotation was carried out by using the BLASTX program within the NCBI nr database and the predicted CDS data as queries. Annotated orthologs of components of the PHO pathway signaling were picked up and their homology to the corresponding *S.* *cerevisiae* PHO genes was reconfirmed on the basis of sequence similarity by using the BLASTP feature in the Saccharomyces Genome Database (http://www.yeastgenome.org/).

### Isolation of APase mutants

An overnight culture was diluted 10,000-fold with sterile water and 0.1 ml of the diluted culture was spread on a YPAD plate. Cells on the plate were irradiated with ultraviolet (20 or 25 J/m^2^) by using a Spectrolinker (TOMY Seiko Co. Ltd., Japan). The plates were then incubated at 28 °C for 2 days. Colonies that appeared on the plates were examined for APase production by the colony staining method (Toh-e et al. 1973). Random integration of linear DNA fragments (van Dijk et al. 2001) was also used to obtain APase mutants of *H.* *polymorpha*. In brief, the plasmid pREMI-Z (a gift from Y. Sakai) was linearized with *Bam*HI and introduced into *H.* *polymorpha* cells. The resultant transformants were selected as zeocin-resistant colonies and their APase phenotype was examined by a colony staining assay. The locus of pREMI-Z insertion was determined by sequencing the plasmid recovered from each transformant.

### Assay of APase activity

APase activity was measured by using intact cells, as described previously (Toh-e et al. 1973). One unit of enzyme activity was defined as the amount of enzyme liberating 1 µmol of *p*-nitrophenol in 1 min at 35 °C.

### Quantitative reverse-transcription PCR analysis

Total RNA was isolated as previously described (van Zutphen et al. 2010), treated with DNase I, and further purified using the RNeasy^®^ Plus Mini Kit (QIAGEN GmbH, Hilden, Germany). A total of 1 µg RNA was used to synthesize cDNA with SuperScript^®^ III Reverse Transcriptase (Invitrogen™, Thermo Fisher Scientific Inc., Waltham, MA, USA) according to the manufacturer’s protocol, and 1 µl cDNA reaction mixture was used in a quantitative PCR reaction with the primers listed in Table S1. PCR products were measured with TaqMan^®^ MGB Gene Expression Kits (Applied Biosystems™, Thermo Fisher Scientific Inc., Waltham, MA, USA) using StepOnePlus™ Real-Time PCR System (Applied Biosystems™). Data were normalized to expression of *HpACT1*.

## Results

### Gene conservation of the PHO pathway in *H.* *polymorpha*

On the basis of the annotated draft genome sequence of BY4329 (Maekawa and Kaneko 2014), we identified genes homologous to regulatory components of the *S.* *cerevisiae* PHO pathway in *H.* *polymorpha* (Table 2). BLASTP alignment was used to estimate amino acid identity between the homologous genes. We found clear orthologs of ScPHO regulatory components in *H.* *polymorpha*—namely, acid phosphatase (encoded by *HpPHO1*), CKI (*HpPHO81*), Pho85–cyclin (*HpPHO80*), CDK (*HpPHO85*), transcription factor (*HpPHO4*), and transcription activator (*HpPHO2*)—as well as genes involved in inositol pyrophosphate synthesis. Therefore, the genomic sequence of *H.* *polymorpha* predicts a signaling architecture in the PHO pathway of this organism similar to that of *S.* *cerevisiae*. In addition, HpPho1 showed 95 % identity to the previously reported Pho1 of *H.* *polymorpha* (ATCC34438; Phongdara et al. 1998).

**Table 2:** Sequence similarity of components of the PHO pathway between *H.* *polymorpha* and *S.* *cerevisiae*

Gene^a^	*Hp* relative to *Sc* ^b^		Length of protein, residues	
	Amino acid identity (%)	Expectation value	*Sc*	*Hp*
*PLC1*	194/478 (40)	5.0e−131	869	897
*ARG82*	115/276 (41)	1.4e−61	355	324
*IPK1*	76/268 (28)	1.5e−12	281	271
*KCS1*	110/224 (49)	8.0e−82	1050	788
*VIP1*	634/942 (67)	0	1146	1103
*PHO81*	157/447 (35)	2.5e−119	1178	1128
*PHO80*	85/171 (49)	1.5e−38	293	293
*PHO85*	195/305 (63)	1.1e−104	302	320
*PHO4*	33/85 (38)	1.5e−10	312	577
*PHO2*	89/225 (39)	1.5e−49	559	439
*PHO1*	155/443 (34)	2.7e−69	467	442

^a^ *H.* *polymorpha* genes except for *PHO1* were designated corresponding to the *S.* *cerevisiae* gene nomenclature. *PHO1* gene has been already named by Phongdara et al. (1998)

^b^Amino acid identity indicates the percentage of amino acid identity between the two species, and the number of amino acids over which the identity was evaluated by BLASTP alignment

The least conserved component between the two yeasts was found to be the transcription factor Pho4. Similar to CgPho4, the most conserved portion of HpPho4 is the C-terminal DNA-binding domain (Fig. S1). Kerwin and Wykoff (2010) reported that CgPho4 induces phosphatase activity independent of CgPho2, probably due to its increased size. Thus, we considered that HpPho4 might possibly be able to drive expression of the PHO promoters in the absence of HpPho2.

The core regulatory system of the PHO pathway consists of four components encoded by *PHO4*, *PHO80*, *PHO81* and *PHO85* (Ljungdahl and Daignan-Fornier 2012). In the following work, therefore, we focused on the respective homologs of *H.* *polymorpha* to determine whether these four genes play the same roles in the PHO pathway as the *S.* *cerevisiae* genes.

### *HpPHO81* is a CDK inhibitor

First, *HpPHO81* was disrupted by insertion of an *HpURA3* fragment. The resulting disruptant showed defective APase production and no derepression of *PHO1* mRNA level under the low-Pi condition (Table 3), which is the same phenotype as observed for disruption of *ScPHO81*. This result indicates that *HpPHO81* is a positive regulator of the PHO pathway at the transcriptional level in *H.* *polymorpha*.

**Table 3:** APase production of *pho* mutants in high- and low-phosphate media

Strain	Relevant genotype	APase activity (mU/mL/OD_660_)		Relative *PHO1* transcript level^a^	
		High-Pi	Low-Pi	High-Pi	Low-Pi
BY4330	Wild type	0.5 ± 0.1*	17.3 ± 0.8*	0.1 ± 0.01*	10 ± 1.7*
H76-1B	*pho81*Δ	0.4 ± 0.1	0.6 ± 0.1	0.7 ± 0.2	0.9 ± 0.7
HPH27-3	*pho80*Δ	23.8 ± 0.3	27.2 ± 1.1	16.5 ± 4.4	32.8 ± 10.2
M1	*pho81*Δ *pho80*-*101*	16.1 ± 1.0	23.3 ± 1.5	ND^b^	ND^b^
YZS216	*pho85*Δ	11.6 ± 0.3	16.0 ± 0.4	7.4 ± 0.9	18.7 ± 2.5
M8	*pho81*Δ *pho85*-*8*	14.4 ± 1.0	23.2 ± 1.4	ND^b^	ND^b^
HPH7-26	*pho4*Δ	0.5 ± 0.04	0.8 ± 0.2	0.6 ± 0.3	0.3 ± 0.06
HPH26	*pho2*Δ	0.6 ± 0.1	13.9 ± 0.6	0.4 ± 0.01	9.9 ± 0.9
YZS153	*pho51*-*7*	13.3 ± 0.8	21.0 ± 1.3	4.5 ± 0.7	8.7 ± 1.0
AP3	*pho53*-*3*	7.9 ± 0.7	21.6 ± 1.3	2.7 ± 0.3	7.2 ± 0.5

* Standard deviation (*n* = 3)

^a^Transcripts of *PHO1* were quantified by RT-qPCR. *PHO1* expression of the wild-type cells grown in low-Pi medium was set as 10. Expression levels of *PHO1* in each mutant are presented as relative ratios to that of the wild type strain under low-Pi condition

^b^Measurement not determined

To determine whether *HpPHO81* function could replace *ScPHO81* function, we constructed an *HpPHO81* expression plasmid (YZ3) for *S.* *cerevisiae*, and transformed it into the *S.* *cerevisiae* strain BY22357 (*Scpho81*∆). The transformant was pre-cultivated in high-Pi medium and inoculated into high-Pi and low-Pi media at an initial OD_660_ of 0.1. APase activity was measured after 23 h of cultivation. As shown in Table 4, the *HpPHO81* gene complemented the defective APase phenotype of the *Scpho81*∆ mutant to a level similar to that of the *ScPHO81* gene, indicating that HpPho81 is a CDK inhibitor. This finding also suggested that a cyclin–CDK complex might function in the PHO pathway of *H.* *polymorpha*.

**Table 4:** Expression of the *HpPHO81* gene complements the *pho81* mutation in *S.* *cerevisiae*

Strain	APase activity (mU/mL/OD_660_)	
	High-Pi	Low-Pi
*Scpho81*Δ + vector (BYP7151)	0.6 ± 0.04*	1 ± 0.1*
*Scpho81*Δ + *HpPHO81* (YZ3)	2.3 ± 0.2	15 ± 0.9
*Scpho81*Δ + *ScPHO81* (YZ6)	2.8 ± 0.2	23 ± 2

* Standard deviation (*n* = 3)

### *HpPHO80* and *HpPHO85* are involved in the regulation of APase expression

**Fig. 1:**
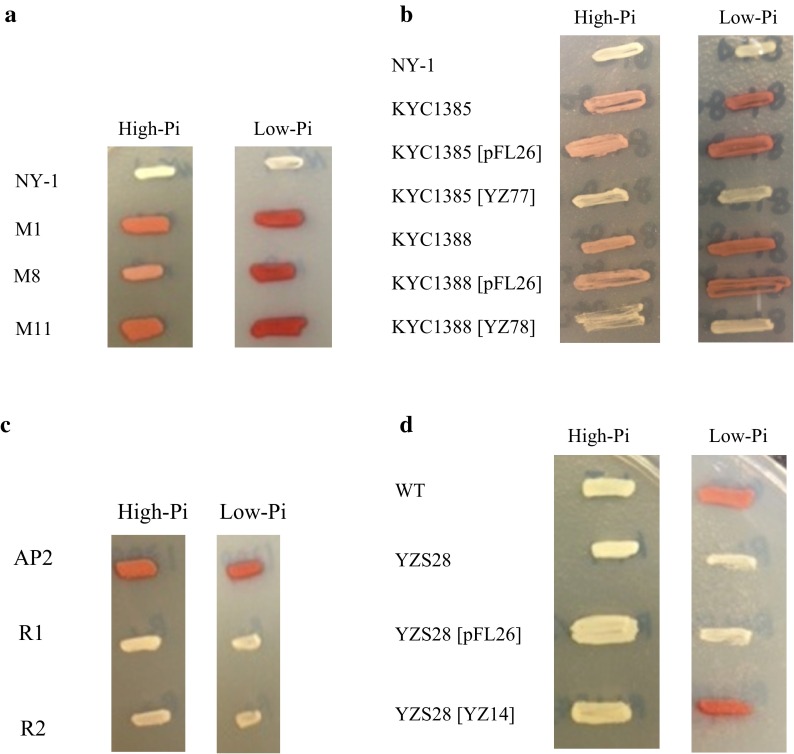
Colony staining assay of APase production in *H.* *polymorpha* mutants. APase activity was determined by a colony-staining assay in high-Pi and low-Pi media. **a** Strains M1, M8, and M11 were isolated by screening mutants obtained after UV mutagenesis of NY-1 (*pho81*∆). **b** Suppressor mutants KYC1385 and KYC1388 were derived from the original suppressor mutants M1 and M8, respectively, and both carried the *pho81*Δ mutation. They were transformed with either the control plasmid (pFL26), or the *HpPHO80* (YZ77) or *HpPHO85* (YZ78) expression plasmid. **c** Strains R1 and R2 were isolated by screening mutants obtained after random integration of pREMI-Z into AP2 (*pho80*). **d** Suppressor mutant YZS28 was derived from the original suppressor mutant R1, and carried the *PHO80* ^+^ gene and the inserted pREMI-Z fragment. YZS28 was subsequently transformed with pFL21 (control) or YZ14 (*HpPHO4*) plasmid

Deletion of either *HpPHO80* or *HpPHO85* resulted in the Pho^C^ phenotype (Table 3), which is the same phenotype observed after deletion of these genes in *S.* *cerevisiae*. Because *pho80* and *pho85* mutations are known to be epistatic to *pho81* mutation in *S.* *cerevisiae* (Toh-e et al. 1973; Ueda et al. 1975), we tried to isolate suppressor mutants from the *Hppho81*∆ strain by UV mutagenesis. Approximately 23,000 colonies were screened for APase activity on both high-Pi and low-Pi plates, and three clones (M1, M8 and M11) exhibited the Pho^C^ phenotype (Fig. 1a). The three isolated strains were each crossed with the strain H76-1B (*Hppho81*Δ), and the resultant diploid hybrids were tested for APase activity. None of the hybrids showed APase activity (i.e., they all had the Pho^−^ phenotype), indicating that the suppressor mutation in each of the isolates was recessive.

Next, the diploids resulting from each cross were sporulated and dissected. Of 9–16 tetrads examined in each case, almost all showed 2 Pho^C^:2 Pho^−^ segregation, indicating that the suppressor mutations each occurred in a single nuclear gene. Using the Pho^C^ segregants, complementation tests were done among the three isolates and the *Hppho80* and *Hppho85* mutants. The results showed that the mutations in M1 and M11 occurred in the same gene, which was allelic to *HpPHO80*, whereas the mutation in M8 was located at the *HpPHO85* locus. Furthermore, the suppressor mutations of M1 and M8 were complemented by the *HpPHO80* (YZ77)- and the *HpPHO85* (YZ78)-expression plasmids, respectively (Fig. 1b). The M1 (*pho81*Δ *pho80*-*101*) and M8 (*pho81*Δ *pho85*-*8*) mutants exhibited levels of APase production similar to those of the null mutants of each gene (Table 3). Thus, it is likely that a cyclin–CDK–CKI system regulates APase expression in *H.* *polymorpha* in a manner similar to *S.* *cerevisiae*.

### Isolation of *Hppho4* as a suppressor of *Hppho80* and *Hppho85*

Next, we constructed *Hppho4*Δ and *Hppho2*Δ strains. As expected, the *Hppho4*∆ strain showed the Pho^−^ phenotype under the low-Pi condition (Table 3). The *Hppho2*Δ strain, however, did not show a defect in the production of APase (Table 3), as has been reported for deletion of *CgPHO2* (Kerwin and Wykoff 2010).

We explored the downstream of *HpPHO80* by screening suppressors of the *Hppho80* mutation (strain AP2; *Hppho80 leu1*) that were generated by random integration of the pREMI-Z plasmid (van Dijk et al. 2001). For unknown reasons, this method did not work well with our strain and many false-positive colonies were obtained after transformation. We therefore replica-plated the first transformants onto fresh YPAD plates supplemented with 200 μg/ml of zeocin before testing APase activity. Among approximately 9100 zeocin-resistant transformants examined, two Pho^−^ mutants (R1 and R2) were isolated (Fig. 1c). Diploids generated between each of these mutants and strain KYC1389 (*Hppho80 ade11*) exhibited the Pho^C^ phenotype, indicating that the two mutations were recessive. Furthermore, analysis of 25 tetrads for each diploid showed 2 Pho^C^Zeo^S^:2 Pho^−^Zeo^R^ segregation, and the two mutations did not complement each other, suggesting that they occurred in the same nuclear gene.

Sequence analysis of zeocin-resistant plasmid DNA recovered from the *Eco*RI-digested genomic DNA of the mutant R1 showed that the pREMI-Z fragment was inserted at the promoter of *HpPHO4* (52-bp upstream of its ATG initiation codon). To examine whether the wild-type *HpPHO4* gene in a plasmid could complement the mutation of R1, we transformed a strain (YZS28) carrying the mutation of R1 with an *HpPHO4* expression plasmid (YZ14). The resultant transformant exhibited the wild-type APase phenotype, as shown in Fig. 1d. Therefore, we concluded that the two suppressors of the *Hppho80* mutation were allelic to *Hppho4*.

We also isolated suppressors of the *Hppho85* mutation by UV mutagenesis of strain KYC1390 (*Hppho85 ura3*). Of approximately 18,000 survival colonies, six suppressor mutants (YZS66, YZS69, YZS72, YZS73, YZS77 and YZS85) were obtained. All diploid cells generated between each mutant and KYC1404 (*Hppho85 leu1*) showed the Pho^C^ phenotype, indicating that all of the suppressors were recessive. Among the diploid strains, those involving YZS72 and YZS73 as representative suppressors were subjected to tetrad analysis. Of 20 or 30 tetrads from the diploids formed between, respectively, YZS73 or YZS72 and KYC1404, the APase phenotype segregated in a 2 Pho^C^:2 Pho^−^ fashion. Furthermore, a complementation test among the six mutants indicated that all suppressors were mutated within the same gene. By backcrossing YZS73 to the wild-type strain BY4330, a Pho^−^ clone without an *Hppho85* mutation (YZS135) was obtained. The strain YZS135 failed to complement the deficient APase phenotype of YZS28 (*Hppho4::pREMI*-*Z*), indicating that the suppressor gene is allelic to the *HpPHO4* gene. Given the results of the mutant analysis and suppressor isolation, we infer that *HpPHO4* encodes a transcription factor required for expression of the APase gene (*PHO1*) under the low-Pi condition, and that HpPho80 and HpPho85 negatively regulate the function of HpPho4.

### Isolation of Pho^C^ mutants

To further explore the PHO pathway of *H.* *polymorpha*, particularly the genes functioning upstream of *HpPHO81*, we screened for mutants showing the Pho^C^ phenotype. The wild-type strain BY4329 was subjected to mutagenesis by UV irradiation, and the colonies resulting on a YPAD plate were transferred to a high-Pi plate, incubated at 37 °C for 1 day, and then tested for APase by the colony staining method. Among approximately 25,000 colonies, 38 mutants showing an unambiguous Pho^C^ phenotype were isolated. The 38 isolated Pho^C^ mutants were then crossed with the wild-type strain BY4330, and the resulting diploids were grown on high-Pi plate and tested for APase activity by colony staining. Four of the 38 mutants were dominant constitutive and the others were recessive constitutive.

For further genetic analysis of the recessive mutants, hybrids generated in the above test were randomly picked up, sporulated and dissected. Among tetrads showing 2+:2− segregation of APase production on YPAD plates, appropriate segregants were chosen and used in complementation tests with other recessive mutants. As a result, the recessive constitutive mutants were divided into three complementation groups, one of which was allelic to *HpPHO80* (11 strains). The remaining two complementation groups were associated with new genes, which were designated *PHO51* and *PHO53*. Mutants with mutations of *PHO51* were most frequently isolated (21 strains).

The four dominant mutants (termed AP4, AP5, C12, and C51) were also subjected to tetrad analysis after crossing with the wild-type strain BY4330. As a result, the Pho phenotype segregated as 2+:2− in each ascus on high-Pi plates, suggesting that the mutations in each of the four mutants are located in a single gene. In the case of *S.* *cerevisiae*, the *ScPHO81*^*C*^ allele has been reported to be dominant constitutive (Creasy et al. 1993; Ogawa et al. 1995). We therefore considered that the present mutants might contain an *HpPHO81*^*C*^ allele. Diploids generated between each dominant constitutive mutant and NY-1 (*Hppho81*∆) were constructed and subjected to tetrad analysis. The Pho phenotype of 8–12 tetrads examined for each diploid showed the 2+:2− segregation pattern on both high-Pi and low-Pi plates and no wild-type recombinant was observed. This observation further suggested that the four dominant constitutive mutants were *HpPHO81*^*C*^ mutants.

Four alleles of *ScPHO81*^*C*^ have been reported to carry the mutation at the N-terminal region of the Pho81 protein (Creasy et al. 1993; Ogawa et al. 1995). To identify the mutation points of the *HpPHO81*^*C*^ alleles, we cloned the alleles of the four *HpPHO81*^*C*^ mutant strains by a PCR-based technique and determined their nucleotide sequences. Notably, the point of mutation of each *HpPHO81*^*C*^ allele differed, being located at not only the N-terminus (S144F in AP4), but also the minimum domain (Y614 N in C12 and F618L in C51) and the C-terminus (N968T in AP5) (Fig. S2).

### *PHO51* and *PHO53* function upstream of *HpPHO81* in the PHO pathway

To determine the position of the *PHO51* and *PHO53* genes in the PHO pathway, we examined the epistatic relationship between the *Hppho81* mutation and the *pho51* or *pho53* mutation. We considered that if *pho51* is epistatic to *Hppho81*, then the *pho51 Hppho81* double mutant would show the Pho^C^ phenotype. In the opposite case—that is, if *Hppho81* is epistatic to *pho51*—the double mutant should show the Pho^−^ phenotype even in the low-Pi condition. We crossed NY-1 (*Hppho81*) and C5 (*pho51*) and performed the tetrad analysis. As shown in Table 5, tetrads exhibited only the 2+:2− segregation pattern of APase production in the low-Pi condition, but 1+:3− (tetratype) and 0+:4− (non-parental ditype) segregations were observed in the high-Pi condition, indicating that *Hppho81* is epistatic to *pho51*.

**Table 5:** Epistasis analysis between the *Hppho81* mutation and the *pho51* or *pho53* mutation

Cross	Segregation of APase phenotype in tetrad				
High-Pi	− − − −	+ − − −	+ + − −	+ + − −	+ + − −
Low-Pi	+ + − −	+ + − −	+ + − −	+ + + −	+ + + +
*Hppho81* × *pho51* (C5)	1	23	5	0	0
*Hppho81* × *pho80* (AP2)	0	0	9	25	5
*Hppho81* × *pho53* (AP3)	2	10	0	0	0

The phenotype was confirmed by staining assay. + and − indicate, respectively, ability and inability to produce APase. C5, AP2 and AP3 are mutants of *PHO51*, *PHO80* and *PHO53*, respectively, whose Pho^C^ phenotype was confirmed to be tightly linked to the mutations by tetrad analysis

In the case of *pho53*, tetrad analysis of diploids generated by crossing with *Hppho81* showed a similar segregation pattern of Pho phenotype. Thus, the results of epistasis analysis with *Hppho81* suggest that both *PHO51* and *PHO53* act upstream of *HpPHO81* in the PHO pathway.

## Discussion

**Fig. 2:**
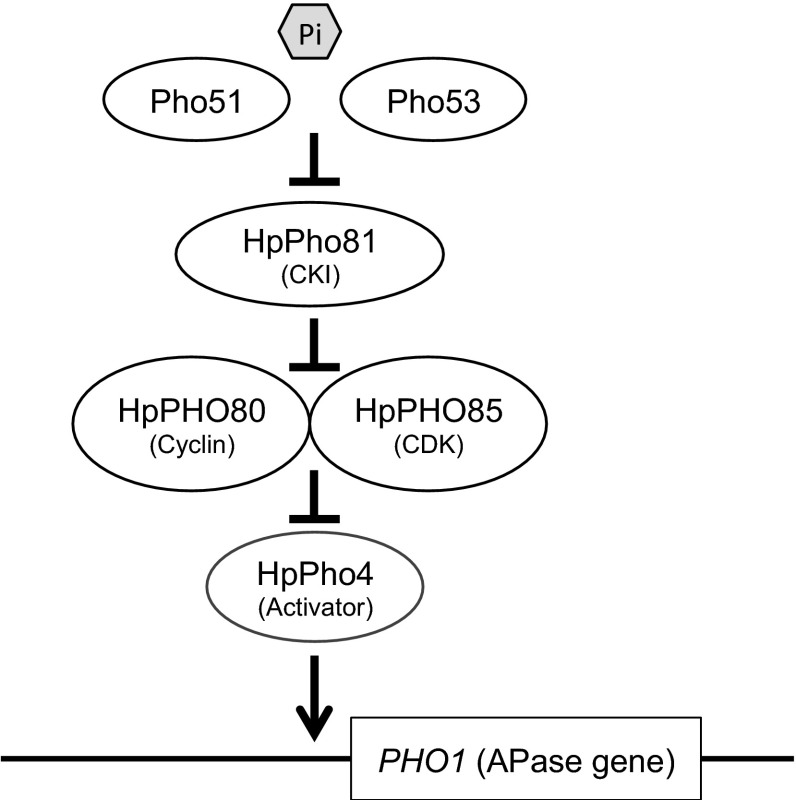
Current genetic interaction model of the PHO pathway in *H.* *polymorpha*. Under the low-Pi condition, the cyclin–CDK (HpPho80–HpPho85) complex is inhibited by the CDK inhibitor HpPho81 and the transcriptional activator HpPho4 induces transcription of the *PHO1* gene. Under the high-Pi condition, the HpPho80–HpPho85 complex phosphorylates and inhibits the function of HpPho4 in the same way as the homologous genes in *S.* *cerevisiae*. In this condition, Pho51 and Pho53 directly or indirectly inhibit the function of HpPho81

The PHO pathway is one of the best model systems for studying the gene regulation network, because substantial information on the function of the regulatory genes in the PHO system of *S.* *cerevisiae* has been accumulated in recent decades. In this study, we have focused on the methylotrophic yeast *H.* *polymorpha*—a species that diverged from *S.* *cerevisiae* more than 100 million years ago (Dujon et al. 2004)—and have characterized its PHO pathway genetically. We confirmed that four genes (*HpPHO81*, *HpPHO80*, *HpPHO85* and *HpPHO4*) are involved in the PHO pathway of *H.* *polymorpha* by comparative genomic analysis and mutant analysis. The *HpPHO81* gene complemented the *S.* *cerevisiae pho81* deletion strain, and several dominant constitutive *HpPHO81*^*C*^ mutations were isolated, similar to those isolated for *S.* *cerevisiae*. The epistatic relationship between *Hppho81* mutation and *Hppho80* or *Hppho85* mutation was also found to be conserved in the PHO pathway of *H.* *polymorpha*. Moreover, *Hppho4* mutation was shown to be epistatic to both *Hppho80* and *Hppho85* mutation. The quantitative real-time PCR analysis of *PHO1* expression under both high-Pi and low-Pi conditions in the relevant mutants indicated that APase (Pho1) production of *H.* *polymorpha* was regulated at transcriptional level by the PHO pathway (Table 3). We therefore propose that the core regulatory components of the PHO pathway are conserved in *H.* *polymorpha* even though it separated from its common ancestor with *S.* *cerevisiae* before duplication of the whole genome during the evolution of ascomycetous yeasts. A schematic of the PHO pathway in *H.* *polymorpha* is shown in Fig. 2.

Through characterization of the PHO pathway in *H.* *polymorpha*, we can infer an evolutionary scheme for development of the phosphate starvation response. Core components of the PHO pathway have been reported to be conserved, or partially conserved, in several species in the phylum Ascomycota. For example, according to Kerwin and Wykoff (2010), the cyclin–CDK–CKI complex is conserved in *C.* *glabrata*, a species that is closely related to *S.* *cerevisiae*. In *N.* *crassa*, the phosphate starvation response involves four regulatory genes—*nuc*-*1*, *preg*, *pgov* and *nuc*-*2* (Kang and Metzenberg 1990; Littlewood et al. 1975; Toh-e and Ishikawa 1971)—corresponding to *ScPHO4*, *ScPHO80*, *ScPHO85* and *ScPHO81*, respectively. Similar to the pathway in *S.* *cerevisiae*, the NUC-2 protein inhibits the function of the PREG–PGOV complex, thereby facilitating translocation of NUC-1 into the nucleus under conditions of Pi shortage (Gras et al. 2009). In *A.* *nidulans*, AnPho80 and AnPho4 play their expected negative role and positive role, respectively, in the PHO pathway; however, the most likely orthologues of ScPho81 and ScPho85 are not involved in this system (Wu et al. 2004). Significantly different from species in the above-mentioned Ascomycota, *S.* *pombe* has no homolog of *ScPHO81* and its closest homologs of *ScPHO80* and *ScPHO85* do not seem to be involved in regulating APase production (Henry et al. 2011). In the phylum Basidiomycota, core components of the PHO pathway are conserved in the species *C.* *neoformans* (Toh-e et al. 2015). Therefore, it seems likely that core components of the PHO pathway existed in the common ancestor of fungi; however, some species have targeted these core components to other types of systems during the course of evolution.

Because the Pho^C^ phenotype of the *pho51* and *pho53* mutants was found to be dependent on *HpPHO81* gene function, we infer that the functional positions of *PHO51* and *PHO53* are located upstream of *HpPHO81* in the PHO pathway (Fig. 2). At present, the function of the *PHO51* and *PHO53* genes remains unclear, and the corresponding genes in the *S.* *cerevisiae* PHO pathway are unknown. Among several recessive constitutive mutations reported in *S.* *cerevisiae*, many are located upstream of *PHO81*, including *PHO84*, *PHO86* and *PMA1*, which are required for function of the phosphate transport system in vivo (Ueda et al. 1975; Lau et al. 1998); *PLC1*, *ARG82* and *KCS1*, which are involved in the synthesis of inositol pyrophosphates; and *ADK1*, which encodes adenylate kinase (Auesukaree et al. 2005). Therefore, it is likely that *PHO51* and *PHO53* might encode proteins involved in phosphate transport or inositol polyphosphate synthesis. In addition, it has been reported that the disruption of either *ACC1*, encoding acetyl-CoA carboxylase, or *PHO23*, encoding a component of the Rpd3L histone deacetylase complex, leads to constitutive APase activity and this phenotype is also *PHO81*-dependent (Lau et al. 1998). Thus, *PHO51* and *PHO53* might encode proteins involved in similar cellular functions. At present, we are trying to characterize these two new genes by bioinformatics analysis of whole-genome sequences, as described by Iida et al. (2014). Furthermore, newly constructed *H.* *polymorpha* YCp-type vectors, which will be described elsewhere, will facilitate both generation of a stable *H.* *polymorpha* genomic DNA library and identification of causative mutations in the near future. Clarification of these mutations might lead to better understanding of the PHO system not only in *H.* *polymorpha*, but also in other yeasts. In addition, it may provide clues to the unsolved problems of the PHO pathway in *S. cerevisiae*.

In *S. cerevisiae*, regulation of the kinase activity of the Pho80–Pho85 complex in response to phosphate concentration is enforced by the Pho81 CKI (Ogawa et al. 1995; Schneider et al. 1994). The Pho81 CKI is constitutively associated with Pho80–Pho85 (Schneider et al. 1994). A small-molecule ligand, IP_7_, interacts noncovalently with Pho80–Pho85–Pho81 and induces additional interactions between Pho81 and Pho80–Pho85 that prevent substrates from accessing the active site of the kinase (Lee et al. 2007, 2008). A particular domain of Pho81—termed the Pho81 minimum domain—is essential for inhibiting both the kinase activity of Pho80–Pho85 toward its Pho4 target and interaction with IP_7_ (Ogawa et al. 1995; Lee et al. 2008). Pho80 has two regions for binding to Pho4 and Pho81 (Huang et al. 2007). Alignment of ScPho81 with HpPho81 shows that the minimum domain is well conserved across the two Pho81 proteins (Fig. S2), suggesting that, similar to ScPho81, HpPho81 might be able to bind to the HpPho80–HpPho85 complex and might be activated by IP_7_. Indeed, the *HpPHO81* gene can function as a CKI in *S.* *cerevisiae pho81*∆ cells, as shown in Table 4. On the other hand, we found that the mutation points of the *HpPHO81*^*C*^ allele were located not only in the N-terminus, but also in the minimum domain and the C-terminus. The mutations in *PHO81*^*C*^ probably result in stronger binding between Pho81^C^ and Pho80–Pho85, making it always win during competition with the wild-type Pho81. Undoubtedly, structural data and detailed biochemical analysis will reveal more clues to the mechanism of Pho80–Pho85 regulation by IP_7_ and Pho81.

Taking all of these observations together, we conclude that the main components of the PHO pathway identified in *S.* *cerevisiae* are conserved in the methylotrophic yeast *H.* *polymorpha,* although these two organisms separated from each other before the occurrence of whole-genome duplication during the course of evolution. Moreover, it seems that the CKI–cyclin–CDK regulatory system is a basic type of the PHO pathway among yeasts.

## Electronic supplementary material

Below is the link to the electronic supplementary material.

Supplementary material 1 (DOCX 20 kb)

Supplementary Fig. S1 Amino acid sequence alignment of Pho4 proteins in three yeast species. The amino acid sequences of the Pho4 protein in *S. cerevisiae* (Sc_pho4), *C. glabrata* (Cg_pho4) and *H. polymorpha* (Hp_pho4) were aligned by Clustal Omega. The known phosphorylation sites of ScPho4 are indicated by boxes (PDF 41 kb)

Supplementary Fig. S2 Amino acid sequence alignment of Pho81 proteins in three yeast species. The amino acid sequences of the Pho81 protein in *S. cerevisiae* (Sc_pho81), *C. glabrata* (Cg_pho81) and *H. polymorpha* (Hp_pho81) were aligned by Clustal Omega. The minimum domain of ScPho81 is denoted by a box. The mutation point in each *HpPHO81* ^*C*^ mutant is indicated by an asterisk (PDF 52 kb)
