# Supplementary material for: Core regulatory components of the PHO pathway are conserved in the methylotrophic yeast Hansenula polymorpha
Source: Curr Genet. 2016 Jan 21;62:595–605. doi: 10.1007/s00294-016-0565-7 (PMC4929164; doi:10.1007/s00294-016-0565-7)
Supplement: Supplementary file 1 — Supplementary material 1 (DOCX 20 kb) [file 294_2016_565_MOESM1_ESM.docx]

Table S1

Primers used in the present study.

| Primer | Sequence* | Purpose |
| --- | --- | --- |
| HpPHO81-1Fnew | 5’-TCGAGCTCGGTACCCGCTTCCAGGACCTCTAATTTCGCT-3’ | Disruption of *HpPHO81* with *HpURA3* |
| HpPHO81-1Rnew | 5’-CTTTACCCAAACTCTAGCTCGTCCGACTCTCTATCTCC-3’ |  |
| HpPHO81-2Fnew | 5’-TGATGTGCTTCGTTGCTTGAGTACGGCGCTGATATCAACC-3’ |  |
| HpPHO81-2Rnew | 5’-CTCTAGAGGATCCCCGGAACCAACTCCAGGGCACTT-3’ |  |
| PHO-URA3F | 5’-AGAGTTTGGGTAAAGCTATTAGGAC-3’ |  |
| PHO-URA3R | 5’-CAACGAAGCACATCAACTGGA-3’ |  |
| HpPHO80-S1F2 | 5’-CTTCTCTCAACTTGCTCTCC-3’ | Disruption of *HpPHO80* with zeocin resistance cassette |
| HpPHO80-S1R2 | 5’-gtcgacctgcagcgtacgTGTGTTTTACCCAGAGAGTT-3’ |  |
| HpPHO80-S2F2 | 5’-cgagctcgaattcatcgatTAACTAGAATTATTTATCAG-3’ |  |
| HpPHO80-S2R2 | 5’-CCGTCGAAGTCCTTAAGTTG-3’ |  |
| HpPHO80-S1sp2 | 5’-GAACGTGGTAGTCCTTTCCG-3’ |  |
| HpPHO80-S2sp2 | 5’-TGTTCACGTACGACAGCTCG-3’ |  |
| HpPHO85-S1 | 5’-ctgatttttttacgaaaaggtgtcatatccagaaaattacagtacgcacttaatttagatATGCGTACGCTGCAGGTCGAC-3’ | Disruption of *HpPHO85* with zeocin resistance cassette |
| HpPHO85-S2 | 5’-ccaccagcttgaaaagctagggcttcaaccgacctcaggagttattaagatcttcaaCTAATCGATGAATTCGAGCTCG-3’ |  |
| HpPHO2-S1F | 5’-TTAAATAATTGCACAGCCGG-3’ | Disruption of *HpPHO2* with zeocin resistance cassette |
| HpPHO2-S1R | 5’-gtcgacctgcagcgtacgCATGTTTTAATTTCGGGAGG-3’ |  |
| HpPHO2-S2F | 5’-cgagctcgaattcatcgatTAGAAGTGCTTGATTTCCGG-3’ |  |
| HpPHO2-S2R | 5’-CTCACGAGCGAGTTGCTTG-3’ |  |
| HpPHO2-S1sp | 5’-GCTATTTATTGGCATTGCGG-3’ |  |
| HpPHO2-S2sp | 5’-TGGATCGGCCAGATCTCGTC-3’ |  |
| HpPHO4-S1F | 5’- GACCTCTGGTCAGTGCCTCG-3’ | Disruption of *HpPHO4* with zeocin resistance cassette |
| HpPHO4-S1R | 5’- gtcgacctgcagcgtacgCATTCCCTGCTGGATTATTT-3’ |  |
| HpPHO4-S2F | 5’- cgagctcgaattcatcgatTAAATGAAAATGTGTAGCTC -3’ |  |
| HpPHO4-S2R | 5’- GCAATTAAACAGCTCGAACG-3’ |  |
| HpPHO4-S1sp | 5’- GCGACAGATGCAAAGACTAC-3’ |  |
| HpPHO4-S2sp | 5’- GCAATTAAACAGCTCGAACG-3’ |  |
| HpPHO81CmpF2 | 5’ CGGTACCCGGGGATCGATCGCAGAGTGGGCCTCA -3’ | Amplification of *HpPHO81^C^* for cloning into the *Bam*HI site of pUC19 |
| HpPHO81CmpR1 | 5’-TGATTACGCCAAGCTGTGACCGTTCCTGGTCTGG-3’ |  |
| InF-ScPhO81-F | 5’-ttaattacaaggatcATGAAATTCGGCAAGTATTT-3’ | Amplification of *ScPHO81* for cloning into the *Bam*HI and *Hin*dIII site of BYP7151 |
| InF-ScPhO81-R | 5' -ccgcggtaccaagctTTACATATCAATATTATTCT-3’ |  |
| InF-HpPhO81-F | 5’-ttaattacaaggatcATGAAGTTCGGCAAGTACTT-3’ | Amplification of *HpPHO81* for cloning into the *Bam*HI and *Hin*dIII sites of BYP7151 |
| InF-HpPhO81-R | 5’-ccgcggtaccaagctTCAGTCTCGTCGTTGTCCTT-3’ |  |
| HpPHO4-pFL26-F | 5'-GGATGACGGGAATAGAGAGGTT-3' | Amplification of *HpPHO4* for cloning into pFL26 |
| HpPHO4-pFL26-R | 5'-TGATCGCATCTCGCGGTGTAAT-3' |  |
| HpPHO80-pFL26-F | 5'-GACAAACAACTTGCCCGCCT-3' | Amplification of *HpPHO80* for cloning into pFL26 |
| HpPHO80-pFL26-R | 5'-ACGCTGAGCGCTGAGTTTCT-3' |  |
| HpPHO85-pFL26-F | 5'-AGGTACCGGGACCTTATTGG-3' | Amplification of *HpPHO85* for cloning into pFL26 |
| HpPHO85-pFL26-R | 5'-CAAGCTTATAGAGCTGCACA-3’ |  |
| PHO1-F | 5’-TCACCGCTGACGACATTACC-3’ | RT-qPCR analysis for *PHO1* expression |
| PHO1-R | 5’-CCTTTGACATTCGTTTCGTAAGC-3’ |  |
| PHO1-probe | 5’-CCATGGGAACCTATTG-3’ |  |
| ACT1-F | 5'-GAGTTGAGAGTTGCGCCAGAA-3’ |  |
| ACT1-R | 5'-GGTTCATTGGAGCCTCAGTCA-3’ |  |
| ACT1-probe | 5’-AGCACCCAGTTTTG-3’ |  |

*Annealing sequences are in upper case
