# Supplementary figures and images for: Core regulatory components of the PHO pathway are conserved in the methylotrophic yeast Hansenula polymorpha
Source: Curr Genet. 2016 Jan 21;62:595–605. doi: 10.1007/s00294-016-0565-7 (PMC4929164; doi:10.1007/s00294-016-0565-7)

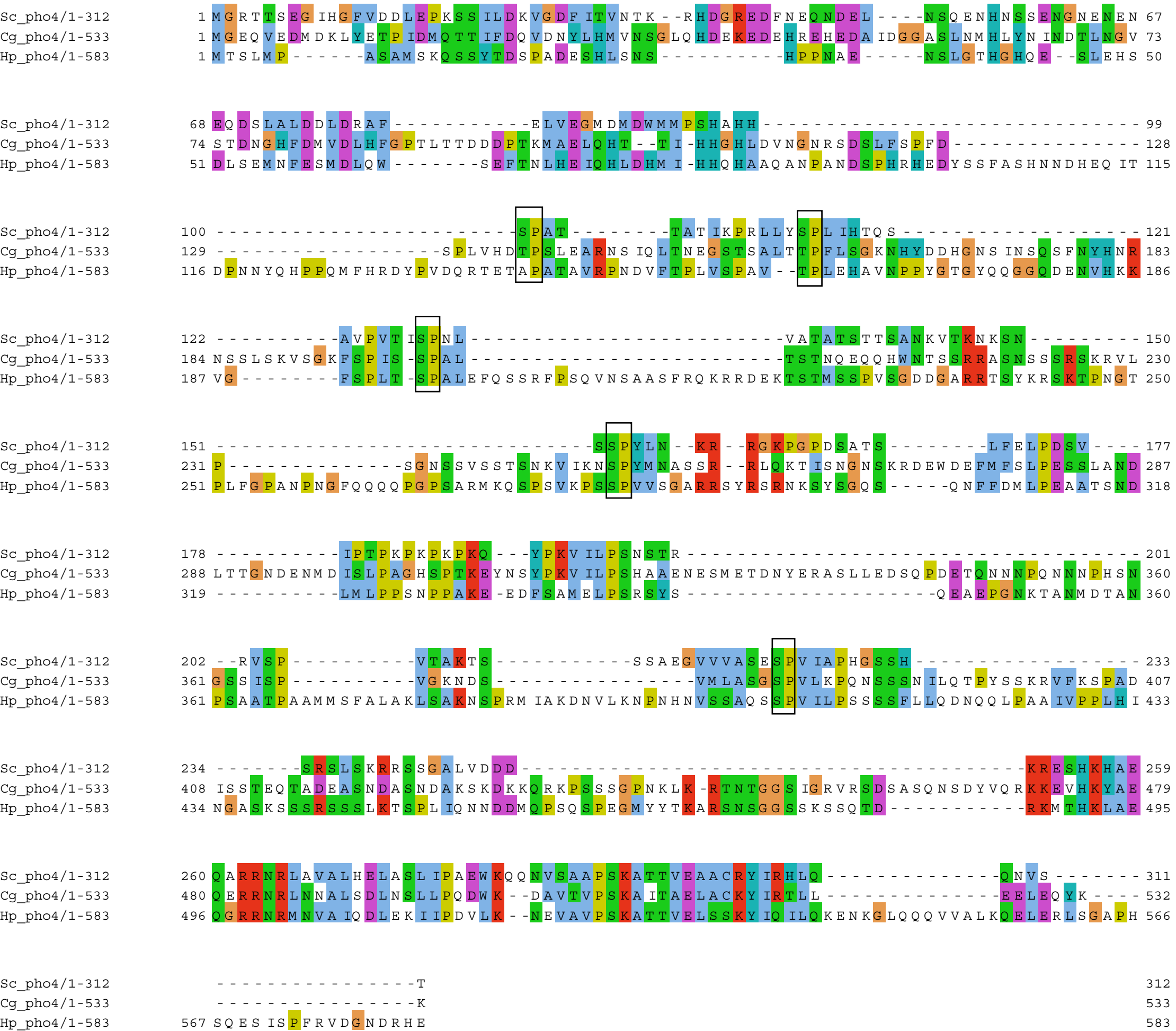

Fig. 1S

Supplement: Supplementary file 2 — Supplementary Fig. S1 Amino acid sequence alignment of Pho4 proteins in three yeast species. The amino acid sequences of the Pho4 protein in S. cerevisiae (Sc_pho4), C. glabrata (Cg_pho4) and H. polymorpha (Hp_pho4) were aligned by Clustal Omega. The known phosphorylation sites of ScPho4 are indicated by boxes (PDF 41 kb) [file 294_2016_565_MOESM2_ESM.pdf]
