# Supplementary material for: Core regulatory components of the PHO pathway are conserved in the methylotrophic yeast Hansenula polymorpha
Source: Curr Genet. 2016 Jan 21;62:595–605. doi: 10.1007/s00294-016-0565-7 (PMC4929164; doi:10.1007/s00294-016-0565-7)
Supplement: Supplementary file 3 — Supplementary Fig. S2 Amino acid sequence alignment of Pho81 proteins in three yeast species. The amino acid sequences of the Pho81 protein in S. cerevisiae (Sc_pho81), C. glabrata (Cg_pho81) and H. polymorpha (Hp_pho81) were aligned by Clustal Omega. The minimum domain of ScPho81 is denoted by a box. The mutation point in each HpPHO81 C mutant is indicated by an asterisk (PDF 52 kb) [file 294_2016_565_MOESM3_ESM.pdf]

```
Sc_pho81     1 MKFGKYLEARQLELAEYNSHFIDYKALKKLIKQLAIPTLKASSDLDLHLTLD----DIDEKIIH 60
Cg_pho81     1 MKFGKYLEARQVELAEYNTHFIDYKALKKLMKQLATVPMINDDDLNASKNLINVDIDFNEASVY 64
Hp_pho81     1 MKFGKYLAARQLELPEYSGYFINYKALKKLINALVANNSND----------------------- 41

Sc_pho81    61 QRLQENKAAFFFKLERELEKVNGYYLARESDLRIKFNILHSKYKDYKINGKLN--SNQATSFKN 122
Cg_pho81    65 RSLQANKASFFFKLERELEKVNLYYVDKESELKVKLDVIVSKMNDYRSSGRLN--SKQAVVYKN 126
Hp_pho81    42 QSLQDKKGSFFFRLERELEKVNNFYLEKESELKFRLDILIEKKNKALSDGRLDNVTKNSIAFVT 105

                                                     *
Sc_pho81   123 LYAAFKKFQKDLRNLEQYVELNKTGFSKALKKWDKRSQSHDKDFYLATVVSIQPIFTRDGPLKL 186
Cg_pho81   127 ISAVIKKFLKDVRNLEQYVELNRTGFAKVLKKWDKRSHSNEKEFYLATVVSVQPIFTRTEVARL 190
Hp_pho81   106 LYDGFKKFSKDLDRLEQFVELNETGFTKVLKKWDKRSKSRTKELYLSTAVNVQPVFHRDEIIEL 169

Sc_pho81   187 NDETLHILLELNDIDNNNRRADIQSSTFTNDDDDDNNTSNNNKHNNNNNNNNNNNNNNNNNNIL 250
Cg_pho81   191 SDEALNLLVDLNDLVEYSVGNGVNSPSTALGPGSSSNAVAISMDGGKNNSNNTKARRSSSS--- 251
Hp_pho81   170 SDLVANNLMELEAKVEGGSFVRYETKEHNKVDDGELPRGDRESDELYTDFYEITAQNANQS--- 230

Sc_pho81   251 HNNYELTTSKISENQLEHLFQASSSSLDMEMEIENWYKEILNIATVKDVQRKHALLRNFRETKI 314
Cg_pho81   252 -----------TGSEKGFFKVLGFSSTDLDLEIEYWIQDIINISTLKDDQRRLATISNFVPTKV 304
Hp_pho81   231 -------------------------KEEQVSKLHEWANQVLSKLSPDSKR--------FTISKV 261

Sc_pho81   315 FTYLLQNSSESFHKNVFSLLKECLTTLFLLLVASPLDDNSLHIFYKSNQDHIDLSYCDEDDQVF 378
Cg_pho81   305 LPRLEELLQNNEQK--EQIEKECITKLFSLLIDSNLVDECLSVVYLACEQYIDFNHIDEDAEIF 366
Hp_pho81   262 FLLLIPN--------------------------LQIPDEALQYFYDNFKEFIDFSLVDDLN--- 296

Sc_pho81   379 SRKNVFHEAASCPEKSRLFILDEALTTSKLSKETVQKLLNAQDIHSRVPLHYAAELGKLEFVHS 442
Cg_pho81   367 SNVNLFHEACACSTAPRSFLLYEALKFKNITSADLKNLMNMKDIHGRIPLHHAAEQGKTEFVEL 430
Hp_pho81   297 -GRTCLHEAASCKTDRVGIVQLCLENNIDP---------TLKDVTGRTCLHYITELGRDDLLLL 350

Sc_pho81   443 LLITN-----LLEDVDPIDSDSKTPLVLAITNNHIDVVRDLLTIGGANASPIEKPILDYSKNVI 501
Cg_pho81   431 ILESN-----LVDMLDIPDQDRKTPLILALESNDIKAVKLLLQHG--------------SNAYP 475
Hp_pho81   351 VLDYAPRIKPLESLIDVVDNESISPLLMAIINNHVSSVEILLKHGAN------------AFPQQ 402

Sc_pho81   502 SSTKVQFDPLNVACKFNNHDAAKLLLEIRSKQNADNAKNKSSQHLCQPLFKKNSTGLCTLHIVA 565
Cg_pho81   476 NAQDKVLDPLSVACKKGNFEAVKLILDFLGDKIEGPSLDK----------------MELLHIVS 523
Hp_pho81   403 SDLKPTYLPLNVACKTGNLEVVQLLLNQASTPAEAKAKGLLTKS-----FQSNAEGLLPLHIVA 461

Sc_pho81   566 KIGGDPQLIQLLIRYGADPNEIDGFNKWTPIFYAVRSGHSEVITELLKHNARLDIEDDNGHSPL 629
Cg_pho81   524 KNSNSSALAELLISKGANVNYGDKFLGRTPLFYAVMNGKDNIVSLLLRCNASIDVMDDEGYTPL 587
Hp_pho81   462 SAG-HDDLIPLLLEYGADINQTDKLNKWTPIFYSVTKGYASTTKKLIEFGANFDIKDEDGFNPL 524

Sc_pho81   630 FYALWESHVDVLNALLQRPLN--------LPSAPLNEINSQSSTQRLNTIDLTPNDDKFDLDIQ 685
Cg_pho81   588 FYTIWESDVKVLNAMLPSIKS--------IKQKKIHSDLLQPKKLTLPNNDLLDLDSSSINDLT 643
Hp_pho81   525 YYALWEGNVGVANVMIESLKASAAAAAGLPPRKELELAPPQAPTLSYTKNVMSPMVPPNRDDLP 588

                                        *   *
Sc_pho81   686 D--------SIPDFALPPPIIPLRKYGHNFLEKKIFIKLKLRPGLESIKLTQDNGIIMSSSPGR 741
Cg_pho81   644 DS-----FENIPAFSLPPPIIPLRKYGHNFLEDKIYVKVIFKAGTESITLENDNDSLIN-APGR 701
Hp_pho81   589 SLSEISNVDMIPDLELPPPIIPLRKYGHNFLEKRIFLKLSFYTNRNSIRLNPDT--FLTSIPGR 650

Sc_pho81   742 ITLSSNLPEIIPRNVILPVRSGEINNFCKDISETNDEEDDDEISEDHDDGEIIFQVDSIDDFSM 805
Cg_pho81   702 IMLSSGSSDIIPRNVLFPVGGTSVTAVDDDLDEE---------------GEIIFYPDTLENFSI 750
Hp_pho81   651 ITISCDKNDLIPRNLLLPVLDNDK--------------------------SITFQTDTFDGFAI 688

Sc_pho81   806 DFEIFPSFGTRIIAKTTAMPFLFKKVAINS----IATMNLPLFDTRLNNIGSLTLDYQIIFPYP 865
Cg_pho81   751 QFDVFPTTGNRLIARTVTPPSFLLSSSMNG----TNSLKIPLVDLKLSVIGTLSVEYQVIQPFS 810
Hp_pho81   689 EFELFPTFGTRLIAKGTLPSSLLQTKCPGVGSKLSGDIEVPLVDLRLRSIGMLRFNYEIVYPYS 752

Sc_pho81   866 GNPLKIINYEPYWKSTG-----SDLMTSSKDGNFVTSSSLNGSFISVLVCALNDETIVAAPKPY 924
Cg_pho81   811 GTSLKVTECEPYWKSTNEEKEPQNSEPSKIDNGYITETSLCGSFETIKIYYLNDGRLVASKEMF 874
Hp_pho81   753 GRPLEISMYDTYWKSSS------AAAATVKSISFVTASSLSGEYYRVKVCYLSDGTPLVCPSWQ 810

Sc_pho81   925 VEFKGTKILLNDLTKEQLEKVVD----------YDFGKIDGSFDEVTLKQYLSSRVVPLRSLLE 978
Cg_pho81   875 VTVNGAKILLMDLTTEQLQAILQS---------SLFIDNEMINTSNDLKILLCKGVFDFKTLLD 929
Hp_pho81   811 ITFNDVVLSVCSFDFDQLSRLLYSAGAFEDLCAQLREVKNLSDDLKKLEDIMAKLYIPLELFLQ 874

Sc_pho81   979 VIPGSAQLVIRVYFP-------------------TDKEIDTIPIKISPFININQFIDKLLLIIF 1023
Cg_pho81   930 LIPSSIKLNIQICFP-------------------TAEEIVSIPVRPSPLIAVDKLINNLLEMIF 974
Hp_pho81   875 IVAVEVSLNLEIFYPSVFELSFFNMKCYSVSAYNLVAKCKSADLSPTTDNVMNNYIDLILNDVF 938

                                             *
Sc_pho81  1024 EHERFLRHSGSGSMRQIVFSSCNWEACSILNWKQPNFPVLLQMKNLLR----DSTTGKFVGDTP 1083
Cg_pho81   975 SNERYLKSTTN-HSRSLVFSSCCWEVCATLNWKQPIFPVFLKFDNLVSYVDPESSTECYKYDTA 1037
Hp_pho81   939 THVRGLRVQGN-RNRSLILSSDNAMVCTILNWKQPNYPVFYNINGIKYNFDTHRFEPCTANGFP 1001

Sc_pho81  1084 NCLKELAVNPQK--------------------MSYLNTELINIHTMVQFAMNNNLLGVTLPYEV 1127
Cg_pho81  1038 HHILDLVQNQEE--------------------YKKL-IKKIDIFGMVKFAIKSHLLGLILPEKL 1080
Hp_pho81  1002 TGSVDVSEAEKPDLKIDNIDSINKDSVVYLNDLQYQDKLTRSIKLGTAFATTNNLLGIIVPNSV 1065

Sc_pho81  1128 LKICPSLARIIKQNGLLLIASVGE------------NDQIPADGGYSGIYYACELLFENNIDM- 1178
Cg_pho81  1081 LHICDTVVDSIRKKGLLVISFSDSP-----------INEAEKELNANGVSDEDEIIFLKSGDDK 1133
Hp_pho81  1066 LSICPDLVKSVRSNGLILVASKDNDETEHAEFGGLLEKSRDHDIDVNGLRFNDILSFKDAIDM- 1128

Sc_pho81       ----
Cg_pho81  1134 NLML                                                             1137
Hp_pho81       ----
```

Fig.2S
